# Low correlation between Ki67 assessed by qRT-PCR in Oncotype Dx score and Ki67 assessed by Immunohistochemistry

**DOI:** 10.1038/s41598-022-07593-7

**Published:** 2022-03-07

**Authors:** Zohair Selmani, Chloé Molimard, Alexis Overs, Fernando Bazan, Loic Chaigneau, Erion Dobi, Nathalie Meneveau, Laura Mansi, Marie-Justine Paillard, Guillaume Meynard, Julien Viot, Marie-Paule Algros, Christophe Borg, Jean-Paul Feugeas, Xavier Pivot, Jean-Luc Prétet, Elsa Curtit

**Affiliations:** 1 Department of Oncobiology, University Hospital of Besançon, Besançon, France; 2 Department of Medical Oncology, University Hospital of Besançon, Besançon, France; 3 Department of Anatomopathology, University Hospital of Besançon, Besançon, France; 4 Bourgogne Franche-Comté University, INSERM, UMR1098, Besançon, France; 5 ICANS, Strasbourg, France; 6 EA3181, UBFC, UFC, Besançon, France

**Keywords:** Breast cancer, Transcription

## Abstract

Breast cancers expressing high levels of Ki67 are associated with poor outcomes. Oncotype DX test was designed for ER+/HER2− early-stage breast cancers to help adjuvant chemotherapy decision by providing a Recurrent Score (RS). RS measures the expression of 21 specific genes from tumor tissue, including *Ki67*. The primary aim of this study was to assess the agreement between Ki67_RNA_ obtained with Oncotype DX RS and Ki67_IHC_. Other objectives were to analyze the association between the event free survival (EFS) and the expression level of Ki67_RNA_; and association between RS and Ki67_RNA_. Herein, we report a low agreement of 0.288 by Pearson correlation coefficient test between Ki67_IHC_ and Ki67_RNA_ in a cohort of 98 patients with early ER+/HER2− breast cancers. Moreover, Ki67_RNA_^high^ tumors were significantly associated with the occurrence of events (*p* = 0.03). On the other hand, we did not find any association between Ki67_IHC_ and EFS (*p* = 0.26). We observed a low agreement between expression level of Ki67_RNA_ and Ki67 protein labelling by IHC. Unlike Ki67_IHC_ and independently of the RS, Ki67_RNA_ could have a prognostic value. It would be interesting to better assess the prognosis and predictive value of Ki67_RNA_ measured by qRT-PCR. The Ki67_RNA_ in medical routine could be a good support in countries where Oncotype DX is not accessible.

## Introduction

In terms of incidence, prevalence and mortality, breast cancer is in first place worldwide in women^1^. The vast majority of patients with breast cancer does not show detectable distant metastasis on diagnosis. Treatment of early breast cancer is based on surgical tumor resection with conditional lymph node dissection. Adjuvant systemic treatments including endocrine or anti-Hormonal therapy (HT) and chemotherapy (CT) aim at the reduction of the distant recurrence rate and improvement of breast cancer specific survival. The decision of adjuvant therapeutic modalities is taken according to several prognostic and/or predictive factors including patient age, tumor size, histological type and grade, lymph node involvement and expression on the tumor of hormone receptors for estrogen (ER) progesterone (PR), and the human epidermal growth factor receptor 2 (HER2) as well as the percentage of tumor cells expressing the nuclear proliferation marker Ki67^2^.

Genomic signatures were designed to give prognostic and predictive information to streamline adjuvant chemotherapy decision in ER-positive, HER2-negative breast cancer patients. Oncotype DX Breast (ODX) is the most widely used molecular signature in this setting and is included in treatment guidelines for estimating both the risk of distant recurrence and predicting adjuvant chemotherapy benefit. ODX measures the RNA of 21 genes (16 cancer-associated genes and 5 housekeeping genes) and uses the expression pattern to calculate a recurrence score (RS) that ranges between 0 and 100^3^. The RS result provides two types of information on tumor biology: (i) prognosis information: an estimate of the individual risk of distant cancer recurrence within 10 years, (ii) predictive information: an estimate of the likelihood of a benefit from chemotherapy^4–6^.

Interestingly in breast cancer, Ki67 RNA (Ki67_RNA_) is a parameter analyzed by several molecular signatures such as PAM50 and ODX^7^. Furthermore, Ki67 is an interesting biomarker in early breast cancer and breast cancers expressing high levels of Ki67 are associated with poor outcomes^8–10^. To provide individualized patient care in the concept of precision medicine, reliability of prognostic and predictive information deriving from Ki67 value is essential^11,12^. Some studies^13,14^ indicate that lowering in Ki67 expression after neoadjuvant endocrine treatment may predict long-term outcome. Nevertheless, substantial variability in Ki67 staining of breast cancer tissue by immunohistochemistry (IHC) and interpretation was found between 30 routine pathology labs. Clinical use of Ki67 staining for therapeutic decisions should be considered with caution and only fully aware of lab-specific reference values^15^. Ki67 staining lacks scoring standardization; various studies have focused on assessment methodology standardization^16^, interobserver reproducibility^17^ and digital image analyses of Ki67 staining^18,19^. However, little is known about variability in IHC Ki67-labelling results between routine pathology labs^20,21^ and its potential influence on interpretation of Ki67 levels in breast cancer. When using Ki67 assessment by IHC in order to consider an indication of adjuvant chemotherapy^22^, clinicians should be aware of the low reproducibility of Ki67 scoring and its questionable analytical validity.

In the present study, we analyzed 98 patients tested by the Oncotype DX Breast from June 2012 to April 2014. For this cohort, Ki67_RNA_ level obtained in patients Oncotype DX signatures were available. The primary aim of this study was to assess the agreement between Ki67_RNA_ and Ki67 staining by IHC (Ki67_IHC_). The other objectives were to analyze the association between the event free survival (EFS) and the expression level of Ki67_RNA_ in ODX signature; and association between RS and Ki67_RNA_.

## Results

### Characteristics of the patient population (Table 1)

**Table 1:** Patient and disease characteristics in the population of ER positive/HER2 negative patients.

	Total (n = 98)	(%)
**Age (years)**		
< 50	34	35
≥ 50	64	65
Mean	57	
**Tumor size (mm)**		
≤ 10	8	8
10 to ≤ 20	57	58
20 to ≤ 50	32	33
> 50	1	1
**Histological type**		
NST	91	93
Other	7	7
**Tumor grade**		
Grade I	61	62
Grade II	28	29
Grade III	9	9
**PR status**		
PR < 10%	18	18
PR ≥ 10%	80	82
**Ki67**_**IHC**_ ** (%)**		
≤ 20	56	57
> 20	39	40
NA	3	3
**Nodal status**		
N0	55	56
Nmic	15	15
N1 (1–3 nodes)	28	29
**Recurrence score**		
Low (< 18)	37	38
Intermediate (18–30)	49	50
High (> 30)	12	12
**Treatments**		
HT	72	73
CT + HT	26	27
**Events**		
No	79	81
Yes	19	19

In this population, 98 women were included from June 2012 to April 2014. All patients were tested by Oncotype DX Breast. The Nodal status was stratified in 3 groups: N0 (node negative), Nmic (micrometastatic) or N1 (1–3 positive nodes). The progesterone receptor status (PR) was considered positive according to standardized European guidelines using a cut-off of ≥ 10% stained tumor cell nuclei. All patients were ER positive and HER2 negative. The Ki67_IHC_ positivity was defined by the rate of > 20% established by St Gallen 2015 recommendations. Almost, The Recurrence score results (RS) are interpreted in three categories (low risk: RS < 18; intermediate risk: RS = 18–30; high risk: RS ≥ 31). The events include the local or metastatic relapse, occurrence of other cancers and death.

*HT* hormonal therapy, *CT* chemotherapy, *NA* not analysed, *NST* no special type.

Complete data sets from 98 breast cancer patients who underwent RS testing were provided from 4 public treatment centers (public hospitals and university hospitals). The included patients were exclusively female and showed a wide age distribution (from 31–81 years) with a mean age of 57 years. The predominant tumor characteristics were no special type (NST) (91%), N0 or Nmic (71%), grade 1 (62%), and tumor size pT1c (1–2 cm) (58%). All patients had ER positive/ HER2 negative tumors. Table 1 shows the patient and disease characteristics of the full population. The RS values were < 18 in 38% (n = 37), 18–30 in 51% (n = 50) and > 30 in 11% (n = 11) of the patients. After surgery and collegial decision, all patients have received a treatment according to the result of ODX test (HT alone or CT-HT) in adjuvant situation.

During the follow-up (57 months), we observed 19 events (19%): three local relapses, ten metastatic relapses (bone, lung, liver and pancreas), three other cancers (contralateral breast, colorectal carcinoma and pancreatic carcinoma) and three deaths.

### Agreement between Ki67_IHC_ and Ki67_RNA_

**Figure 1:**
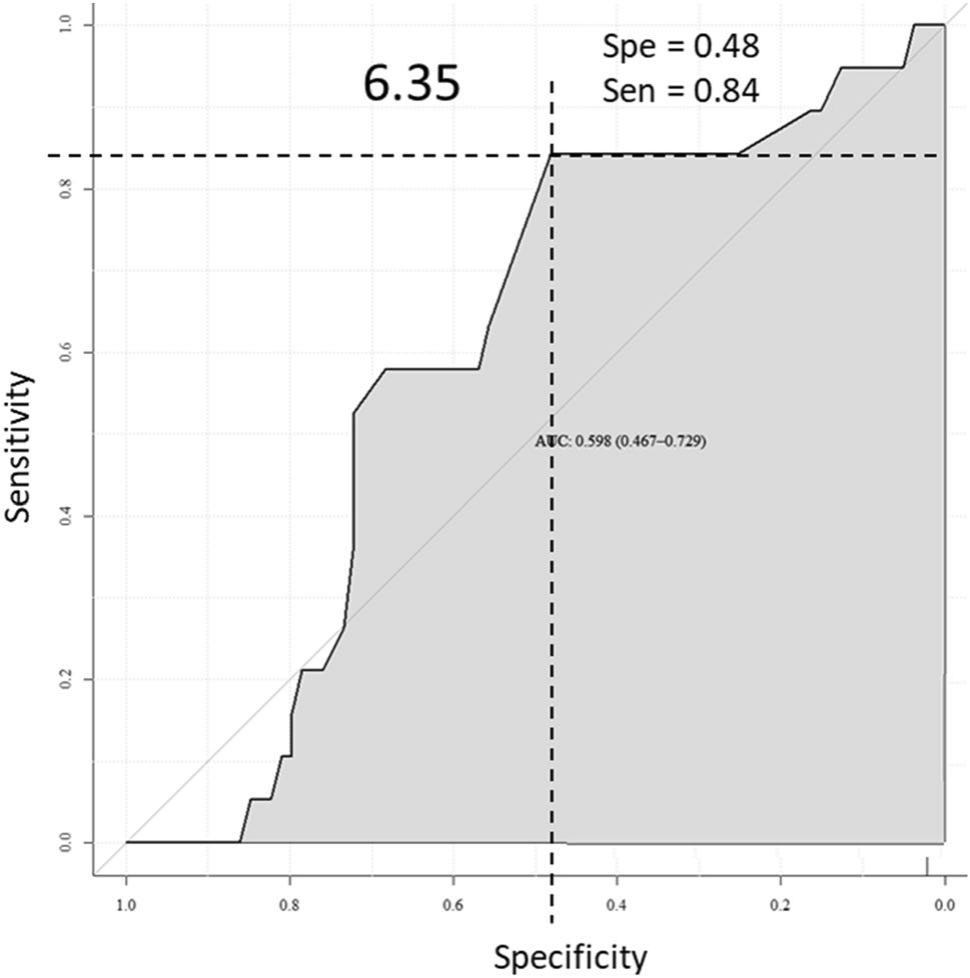
Threshold determination, specificity and sensitivity of Ki67_RNA_. Threshold of Ki67_RNA_ was determined using package pROC version 1.16.1^44^. An optimal threshold of Ki67_RNA_ at 6.35 units was found with a specificity of 48% and sensitivity of 84%.

The Ki67_IHC_ positivity rate of > 20%^23^ was used to define for the “high-risk” tumor group. We showed by ROC curve analysis an optimal threshold for Ki67_RNA_ at 6.35 with a specificity of 48% and a sensitivity of 84%. With this cut off, the Ki67_RNA_^high^ were ≥ 6.35 and Ki67_RNA_^low^ were < 6.35 (Fig. 1). A correlation between Ki67_RNA_ expression and Ki67_IHC_ score was found (R = 0.288**;** *p* = 0.0047) (Supplementary data 2). In the cohort, 29 tumors were Ki67^high^ and 29 were Ki67^low^ by the two methods, which represent a match between the results in 58 patients (61%) (Table 2). Discordances were observed in 37 patients (39%): 10 samples were Ki67_IHC_^low^/Ki67_RNA_^high^ and 27 samples were Ki67_IHC_^high^/Ki67_RNA_^low^.

**Table 2:** Agreement between Ki67_RNA_ and Ki67_IHC_ (n = 95).

	Ki67_RNA_^High^	Ki67_RNA_^Low^
Ki67_IHC_^High^	29 (30.5%)	27 (28%)
Ki67_IHC_^Low^	10 (11%)	29 (30.5%)

Pearson’s correlation coefficient equal to 0.288 (p = 0.0047) indicate a significant low degree of agreement.

### Events-free survival among all patients according RS groups and Ki67 status

**Figure 2:**
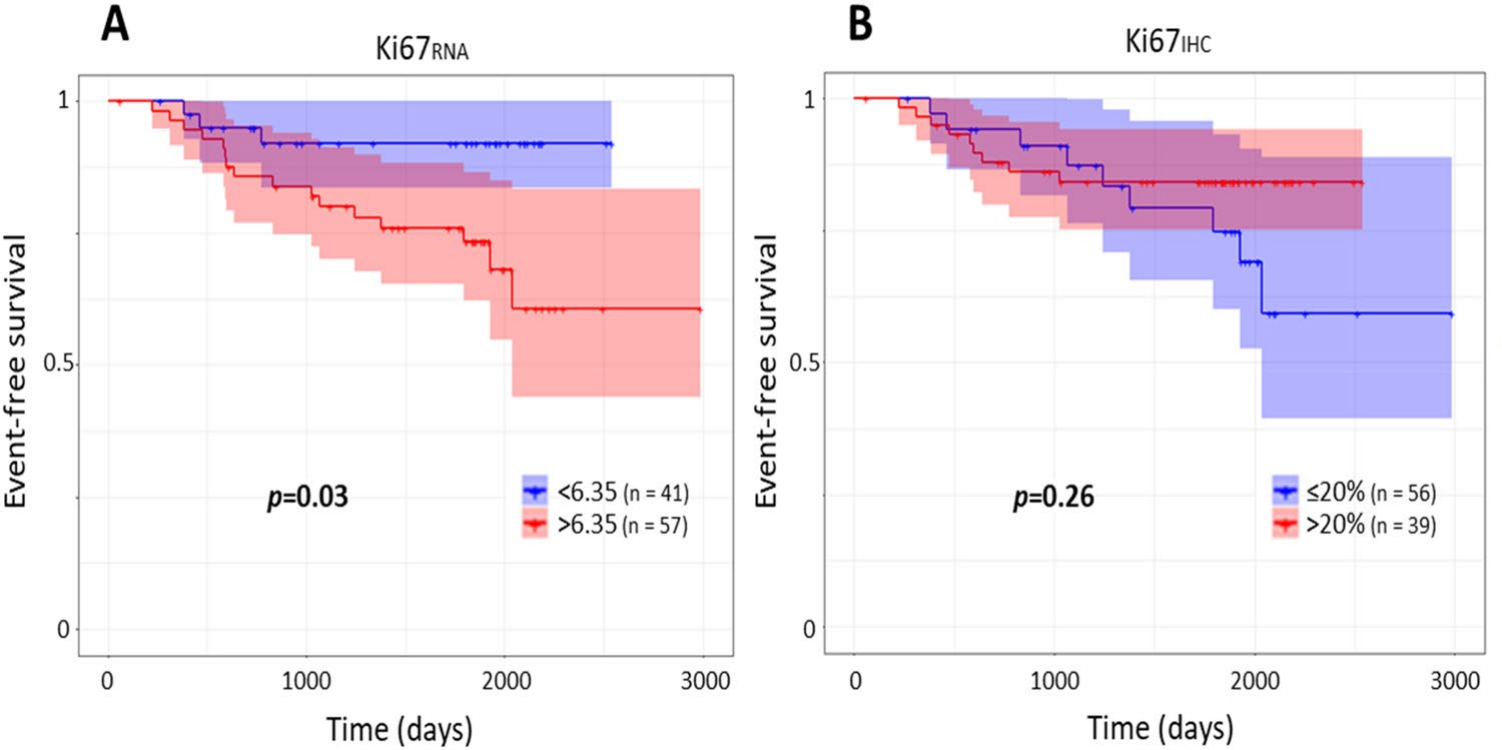
Events-free survival according Ki67 status by ODX test and by immunochemistry. The survival follow-up was analyzing by the package survival version 3.1-8^45,46^. The events include the local or metastatic relapse, other cancers occurrence and death with a mean follow-up of 57 months. (**A**) Ki67_RNA_ were analyzed according to two groups using the 6.35 units’ threshold. (**B**) The Ki67_IHC_ were analyzed according to two groups using the rate of > 20%. Statistical analysis were obtained by a Cox univariate test.

**Figure 3:**
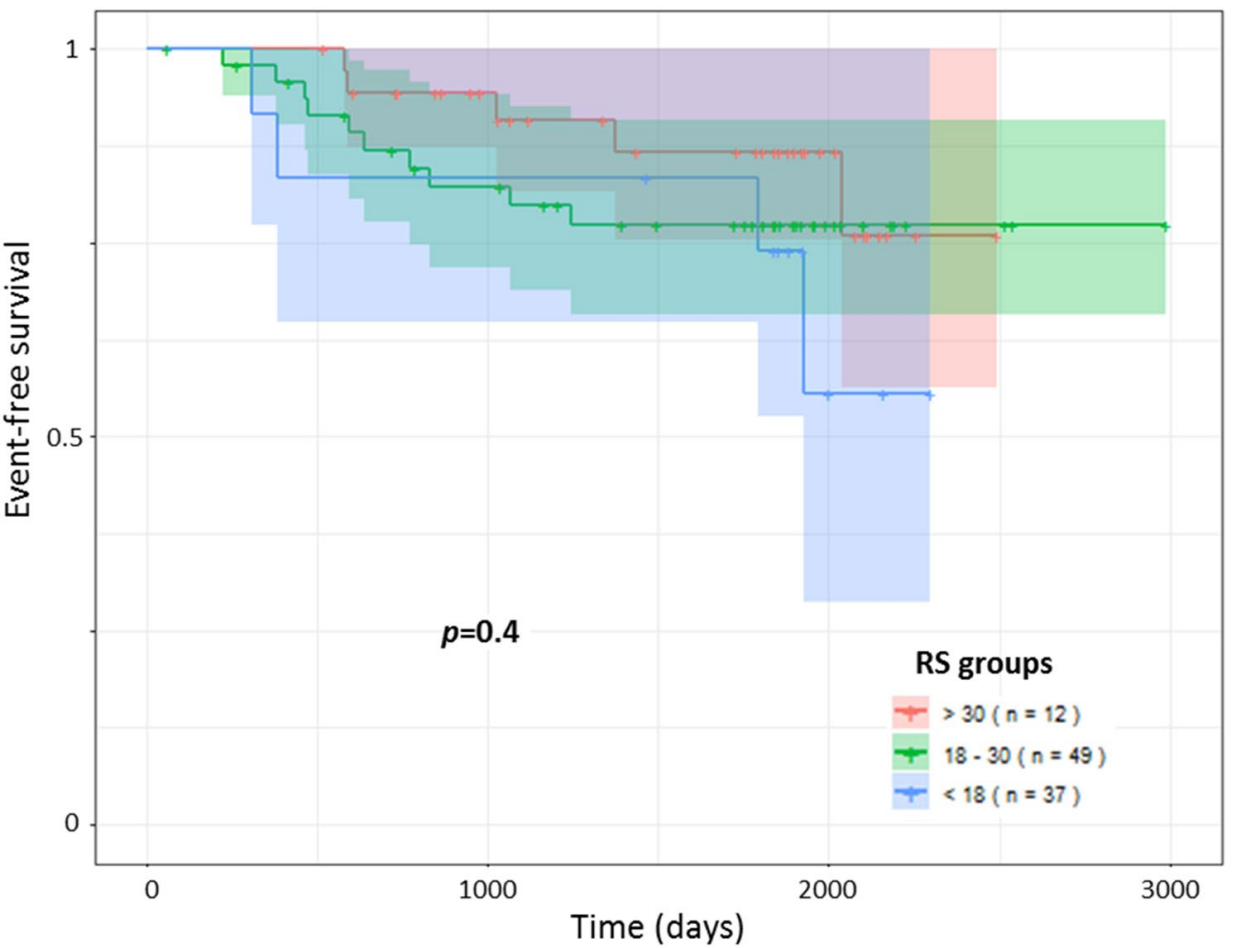
Events-free survival among all patients according RS groups. The survival follow-up was analyzed by the package survival version 3.1-8^45,46^. The events include the local or metastatic relapse, other cancers occurred and death with a mean follow-up of 57 months. The RS are interpreted in three categories (low risk: RS < 18; intermediate risk: RS = 18–30; high risk: RS > 30). Statistical analysis were obtained by a Cox univariate test.

A significant relationship was observed between EFS and the level of Ki67_RNA_ expression (*p* = 0.03) (Fig. 2A) unlike Ki67_IHC_ (*p* = 0.26) (Fig. 2B). In univariate Cox analysis, we observed a hazard ratio of 3.92 [1.14–13.49] for Ki67_RNA_ and 0.57 [0.263–1.48] for Ki67_IHC_. The same difference was observed in multivariate analysis (Table 3). However, we did not observe any significant difference on EFS according to the three RS groups risk (*p* = 0.4) (Fig. 3).

**Table 3:** Cox proportional hazards regression of EFS according Ki67 status.

	Cox, univariate	Hazard ratio, univariate [IC95]	Cox, multivariate	Hazard ratio, multivariate [IC95]
Ki67_IHC_	p = 0.26	0.57 [0.263–1.48]	p = 0.11	0.46 [0.18–1.12]
Ki67_RNA_	p = 0.030	3.92 [1.14–13.49]	p = 0.033	3.93 [1.12–13.82]

Using the age categories from the TAILORx study^24^, the association between EFS and Ki67_RNA_ status, was found only for women under 50 years (*p* = 0.01) and not for women over 50 years (*p* = 0.4) (Supplementary data 3). No association between Ki67_IHC_ and EFS was observed whatever the patient age (*p* > 0.20).

### Ki67_RNA_ association with RS

**Figure 4:**
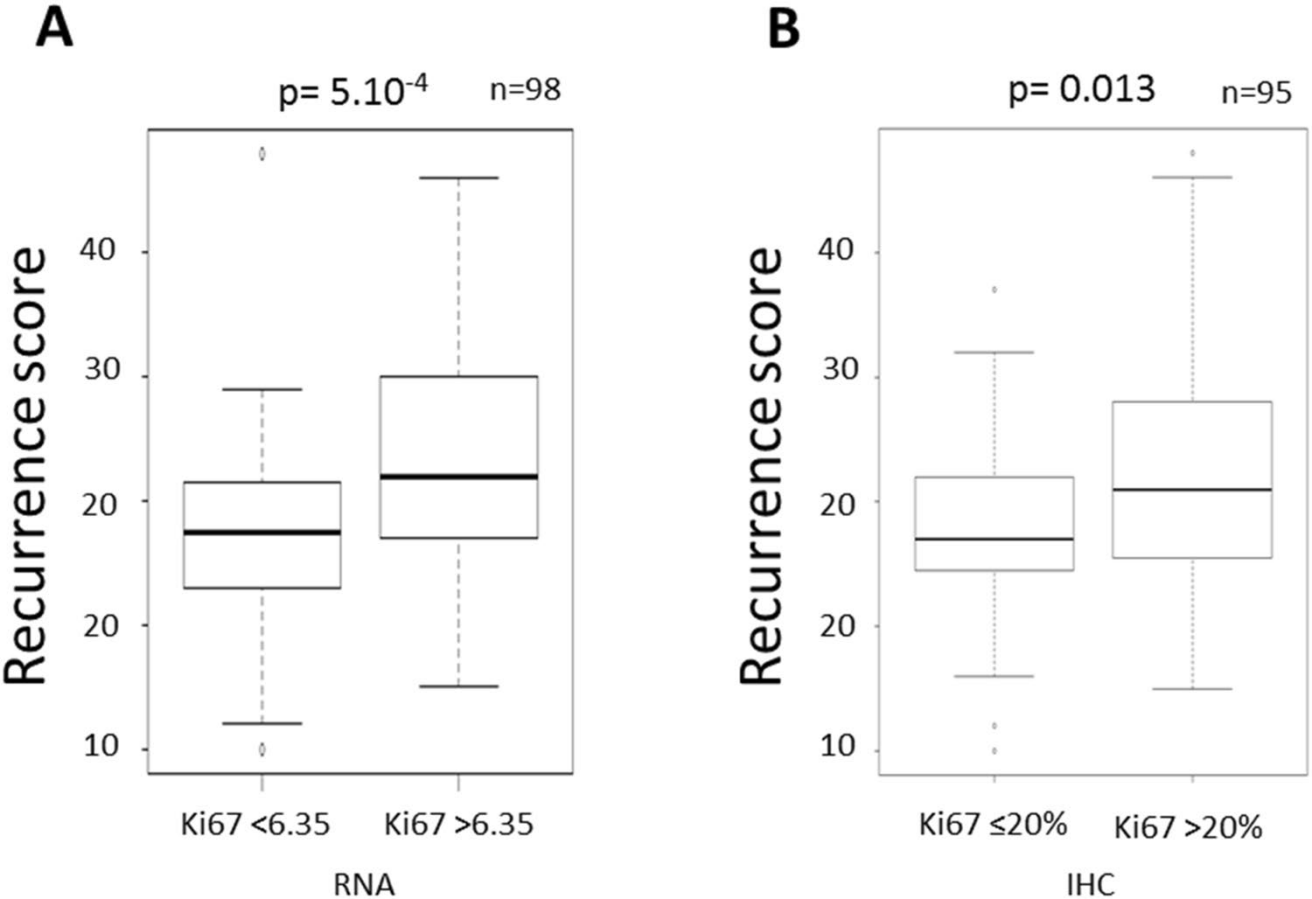
Association between RS and Ki67 status. (**A**) The association of RS with Ki67_RNA_ were analyzed according to the 6.35 units’ threshold. (**B**) The association of RS with Ki67_IHC_ were analyzed using the rate of > 20%.

As expected, Ki67_RNA_ levels were significantly associated with RS (*p* = 5 × 10^−4^) (Fig. 4A). To a lesser extent, an association between ODX test with Ki67_IHC_ status was also observed (*p* = 0.013) (Fig. 4B). Supplementary data 4 shows the distribution of Ki67_IHC_ score and Ki67_RNA_ level according to patient age, tumor grade and size, PR status, nodal status, Recurrence score, treatment and events.

## Discussion

In this study, we focused on the Ki67_RNA_ expression level of 98 ER-positive, HER2-negative breast cancer with Oncotype DX signatures. The primary aim of this study was to assess the agreement between Ki67_RNA_ and Ki67 staining by IHC (Ki67_IHC_). Outcomes and tumors characteristics were also assessed.

### Study population

The majority of our data are consistent with the PONDx real life study^25^ which found a female population over 50 years (70%) with NST tumors (78%) ranging between 2–5 cm (89%), grade II (68%) and without lymph node involvement in 79% of cases. The PONDx cohort included more grade II than in our population (68% vs 29%) with a higher contingent of lobular carcinoma (13% vs 7%). In PONDx, RS results by prognostic category used between 2012–2014 were: < 18: 54%, 18–30: 36%; > 30: 10%. Compared to this real-life study, the local cohort shows an inversion of the proportions between populations at low risk (38% vs 54%) and those at intermediate risk (51% vs 36%).

The few differences observed in the distribution of the risk groups established by the ODX test can be explained by an over-representation of grade II tumors in the PONDx study. However, our cohort remains comparable to this larger population for most other demographic parameters.

### Differences between Ki67_RNA_ and Ki67_IHC_

Comparisons of Ki67 status showed a discrepancy between the two different types of evaluation. Indeed, half of Ki67_RNA_^high^ (> 6.35) were found Ki67_IHC_^low^ (Table 2). The observed correlation between the two techniques, is therefore weak even if significant (R = 0.288, *p* = 0.0047) (Supplementary data 2). Technically, IHC shows greater variability^20,21^ and is less reproducible than the quantification of mRNAs. Another study comparing microarray data with IHC confirm in a series of 520 samples^26^. These results are in contradiction with those observed by Finsterbusch et al.^27^*.* These authors found a strong correlation between Ki67 in IHC and mRNA level of Ki67 obtained by Mammatyper in 101 patients. In this retrospective study, their method of assessing Ki67 protein expression was different from ours. On the other hand, all the contradictory data around its interpretation highlight the lack of reproducibility and the variation between observers.

The prognostic value of Ki67 in IHC is now no more debated because it was demonstrated in numerous publications^10,28^. These two meta-analyses of Stuart-Harris et al. and Azambuja et al*.* have confirmed the prognostic information on 44 and 46 studies but the threshold were comprising between 0–34% of Ki67 positive cells by anti-Ki67 or anti-MIB-I antibodies. Currently, there is no standardization for the interpretation of the Ki67 status by IHC^16^. As a consequence, it generates inter-site and inter-observer variability within the same site^17^. Recently, Nielsen et al. have updated the recommendations from the International Ki67 in Breast Cancer Working Group^29^. These authors provide solutions for a pre-analytical standardization of Ki67_IHC_. However, they specify that the Ki67_IHC_ should be used to drive patient care only when 5% or less or 30% or more cells are positive due to poor reproducibility between observers^29,30^. A single-center prospective study with double-blind reading seems more suitable in order to avoid interpretation bias. In contrast, the Oncotype DX test which is a molecular based and standardized test provides objective results that are independent of the observer.

Furthermore, the ODX test analyzes the level of Ki67 messenger RNAs in the tumor sample. This analysis therefore considers the transcriptional status of the *Ki67* gene level not only in tumor cells but also in associated cells such as lymphocytes, stromal or endothelial cells. Therefore, it is an overall estimate of Ki67 expression. By IHC, the protein expression is scored only on tumor cells. The two methods do not provide the same types of information on Ki67 status. These two types of evaluation of Ki67 are probably complementary because the Ki67_IHC_ gives informations about the tumor specific proliferation index and the Ki67_RNA_ would reflect the proliferation of the tumor and its microenvironment. For all the reasons mentioned above, the association between RS and Ki67_IHC_ status is lower but remains significant (*p* = 0.013).

### Events free survival analyses

On the one hand in this study, adaptation of treatment based on RS permit to obtain equivalent EFS in populations with low and high risk of recurrence. Indeed, the lack of association between RS and EFS can be explained by the treatment (HT alone or CT-HT) performed according the RS interpretation based on Paiks et al. retrospective study^31^. Indeed, during the inclusion period, we used the Paiks et al. criteria for treatment as follow: low risk (RS < 18) patients were treated by HT alone and high risk (RS > 30) patients by CT-HT. The treatment for intermediate risk (RS = 18–30) patients was assigned on a case-by-case basis. The clinical validity and utility of the RS has been demonstrated prospectively across multiple studies in breast cancer patients worldwide including multiple validation studies as well as long-term prospective studies (TAILORx^24^, WSG Plan B^32^ and analyses from a prospective epidemiological database^33^). Based on level Ia evidence, the Oncotype DX test has been incorporated in leading internationally-accepted clinical guidelines on the treatment of early breast cancer (St. Gallen^34^, ESMO^35^, and ASCO^36^). This therapeutic adaptation will therefore smooth the differences between the categories of the ODX test and at the same time demonstrates the value of the information provided by its score.

On the other hand, we were able to show that Ki67_RNA_^high^ was significantly associated with the occurrence of events (*p* = 0.03) but we did not find any association between Ki67_IHC_ and EFS (*p* = 0.26).

In univariate analysis of Ki67_RNA_, we obtained a hazard ratio of 3.92 [1.14–13.49] and performed a power calculation under the Cox proportional-hazards model and found a power of 0.82. The significant of our study is near the decisional threshold for clinical studies. Further investigations will be required to confirm these results.

### Correlation between Ki67_RNA_ and RS

As expected in our cohort, the RS obtained is strongly associated with the Ki67_RNA_ (*p* = 5 × 10^−4^) which is one of the components of the ODX test (Fig. 4A). To optimize RS interpretation, the TAILORx subgroup study shows a benefit of CT in women ≤ 50 years old with an RS between 16 and 25 (*p* = 0.004). CT could be avoided in women over 50 years old with a RS < 26, in women 50 years old or less with an RS < 16^37^. Recently at San Antonio Breast cancer symposium, the results of RxPONDER study were presented^38^. Now, postmenopausal women with 1–3 positive nodes and RS 0–25 can safely forego adjuvant CT without compromising invasive disease-free survival. The premenopausal women with positive nodes and RS 0–25 likely significantly benefit from chemotherapy^38^.

Oncotype DX Breast enables relevant net reductions in chemotherapy use, sparing patients from serious toxicities^7^. On this other side, its clinical impact and pharmacoeconomic benefit in routine care have been shown in 20 decision-impact studies^39–41^. However, in many places, the oncotype score remains inaccessible or not reimbursed^7,25,41^. This is why the evaluation of Ki67_RNA_ level by molecular biology techniques^42,43^ could be a low-cost prognostic alternative in some countries. However, this hypothesis will have to be validated during a prospective translational study.

In conclusion, we observed of low agreement between Ki67_RNA_ tumor level measured by qRT-PCR and Ki67 protein labelling by IHC. Substantial variabilities in Ki67_IHC_ of breast cancer tissue and interpretation have been shown and widely published. The clinical use of Ki67 labelling should be cautious and limited by the low reproducibility of Ki67 scoring and its questionable analytical validity.

Unlike Ki67_IHC_ and independently of the RS, Ki67_RNA_ could have a prognostic value. The assessment of Ki67_RNA_ by qRT-PCR on breast cancer tumor would be feasible and cost effective. It would be of great clinical utility to better assess the prognosis and predictive value of Ki67_RNA_. Presentation of Ki67 status in Genomic Health report could also be helpful for therapeutic decisions on borderline situation.

## Methods

### Ethics statement

All procedures performed in studies involving human participants were in accordance with the ethical standards of the national research committee and with the 1964 Helsinki Declaration and its later amendments. In France, this search is considered like a non-interventional study according to European legislation. All patients were individually informed that their data should be used for scientific research. Informed and written consents were obtained from all subjects, or if subject are deceased, from a parent and/or legal guardian. All experimental protocols were approved by the « *comité de protection des personnes* » (CCP) of Besançon, France.

### Patients and tumors characteristics

Our study was an observational multicenter retrospective study collecting data on the real-life use of Oncotype DX Breast Recurrence Score test by physicians in clinical practice settings in France. Patients eligibility criteria for this analysis correspond to the population for which the Oncotype DX Breast Recurrence Score test is validated, i.e. adult patients with a recent first diagnosis of a single early invasive breast tumor with ER+/HER2− status, plus available documentation of lymph node involvement as either N0 (node negative), Nmic (micrometastatic) or N1 (1–3 positive nodes). The following data were documented: patient age and sex, conventional clinical and pathological disease characteristics including histologic type, tumor size and grade, nodal status, receptor status including ER, PR, and HER2, and the Ki67 proliferation marker by IHC. Between 2012 and 2014, the RS results were interpreted in three categories (low risk; intermediate risk; high risk with two cut-offs: 18 and 30). The event free survival (EFS) includes the local or metastatic relapse, other cancers and death with a mean follow-up of 57 months. Following the guidelines, the Oncotype DX Breast Recurrence Score test was realized after surgery and adjuvant treatments were adapted according to the RS. The interpretation of RS values based on Paiks et al. study^31^ and the associated treatment (hormonal therapy plus/minus chemotherapy, or other modalities) were also collected.

### Immunohistochemistry analyses

The hormonal receptor status (ER and PR) was considered positive according to standardized European guidelines using a cut-off of ≥ 10% stained tumor cell nuclei. The Ki67_IHC_ evaluation was realized in 2 publics and 2 private pathological departments. In these structures, the Ki67_IHC_ was assessed with monoclonal antibody MIB1, based on the recommendations from the International Ki67 in Breast Cancer Working Group^16^. Indeed, Ki67_IHC_ is a nuclear staining and is evaluated regardless the staining intensity. Ki67_IHC_ was defined as the percentage of positively stained tumor cells among the total number of tumor cells scored. For each cases, at least 3 randomly selected high power (40 × objective) fields were scored^16^. The Ki67_IHC_^high^ corresponding to Ki67 > 20% were defined “high-risk” subpopulations of tumors according to the recommendations established by St Gallen in 2015^23^. In 2019, the ESMO clinical practice guidelines for diagnosis, treatment and follow-up in early-stage breast cancer validated this threshold, while presenting its limits^30^.

### Ki67_RNA_ threshold determination and events statistical analyses

Descriptive statistics were used to summarize clinicopathological characteristics. Variables were described by the size and rate. For each of the 98 patients of our cohort, Genomic Health, Inc. provided normalized unit values of Ki67_RNA_ expression. Based on 5 housekeeping genes expressions (*GAPDH, RPLPO, GUS, TFRC, β-actin*), the Ki67 RNA expression is normalized in Oncotype DX test. These values were distributed according to a Gaussian curve in our cohort (Supplementary data 1).

We have determined the optimal threshold using ROC curves approach with the point maximizing the area under ROC curve with R package pROC version 1.16.1^44^. The quantitative values of IHC and RNA were turned in clinically used qualitative variables. The statistical comparison between clinically used qualitative IHC and RNA was realized by Pearson coefficient. The statistical comparison between quantitative IHC and RNA was realized by the Pearson correlation coefficient. Statistical analysis were performed using R version 3.6.2^45^. The survival follow-up was analyzed with the Cox test on Kaplan–Meier estimates using the package survival version 3.1-8^46^.

## Supplementary Information

Supplementary Information.

## Data Availability

The datasets generated and analyzed during this study (birthdate, admission date, discharge date, date of death…), are available from the corresponding author on reasonable request.
