# Supplementary material for: Low correlation between Ki67 assessed by qRT-PCR in Oncotype Dx score and Ki67 assessed by Immunohistochemistry
Source: Sci Rep. 2022 Mar 7;12:3617. doi: 10.1038/s41598-022-07593-7 (PMC8901910; doi:10.1038/s41598-022-07593-7)

ADDITIONAL FILES
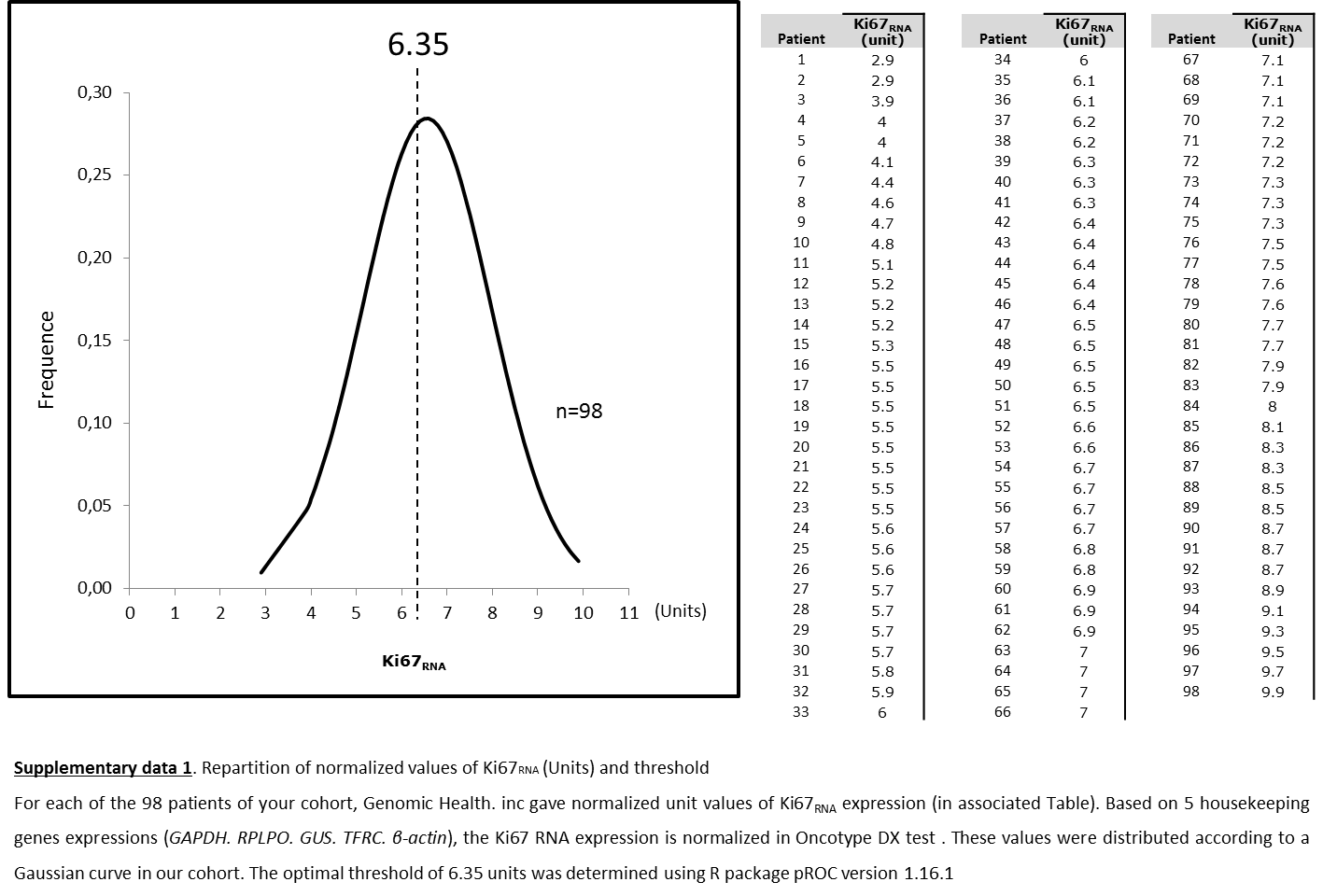


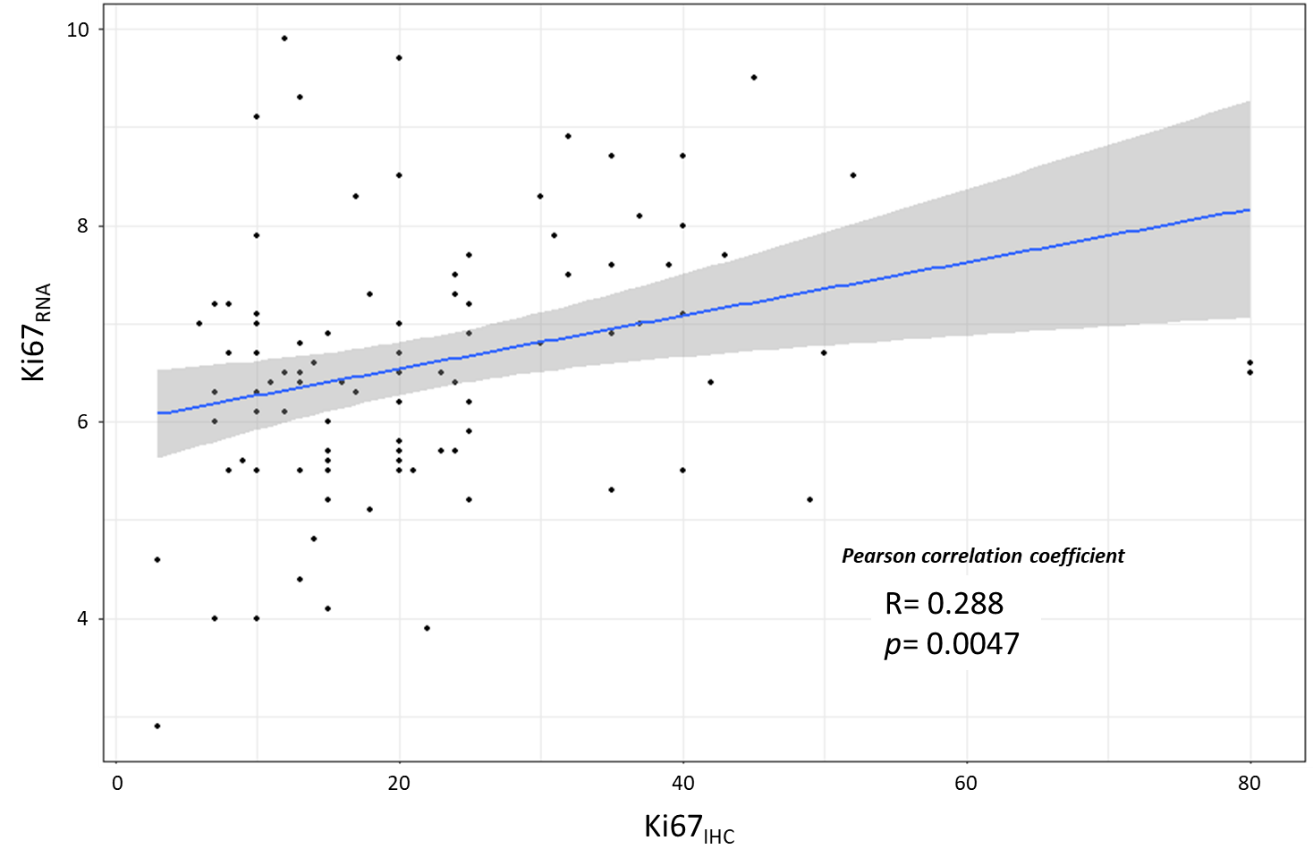


**Supplementary data 2.** Correlation between Ki67_RNA_ and Ki67_IHC_


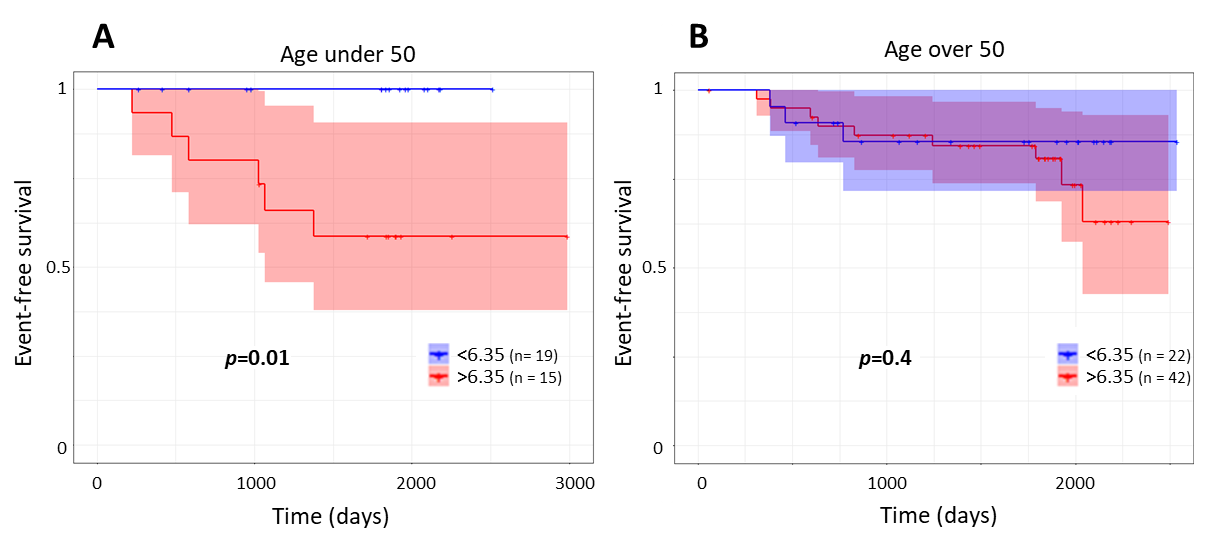


**Supplementary data 3.** EFS according to age subgroups and their respective Ki67_RNA_ status

Statistical analyses were obtained by a Cox univariate test.


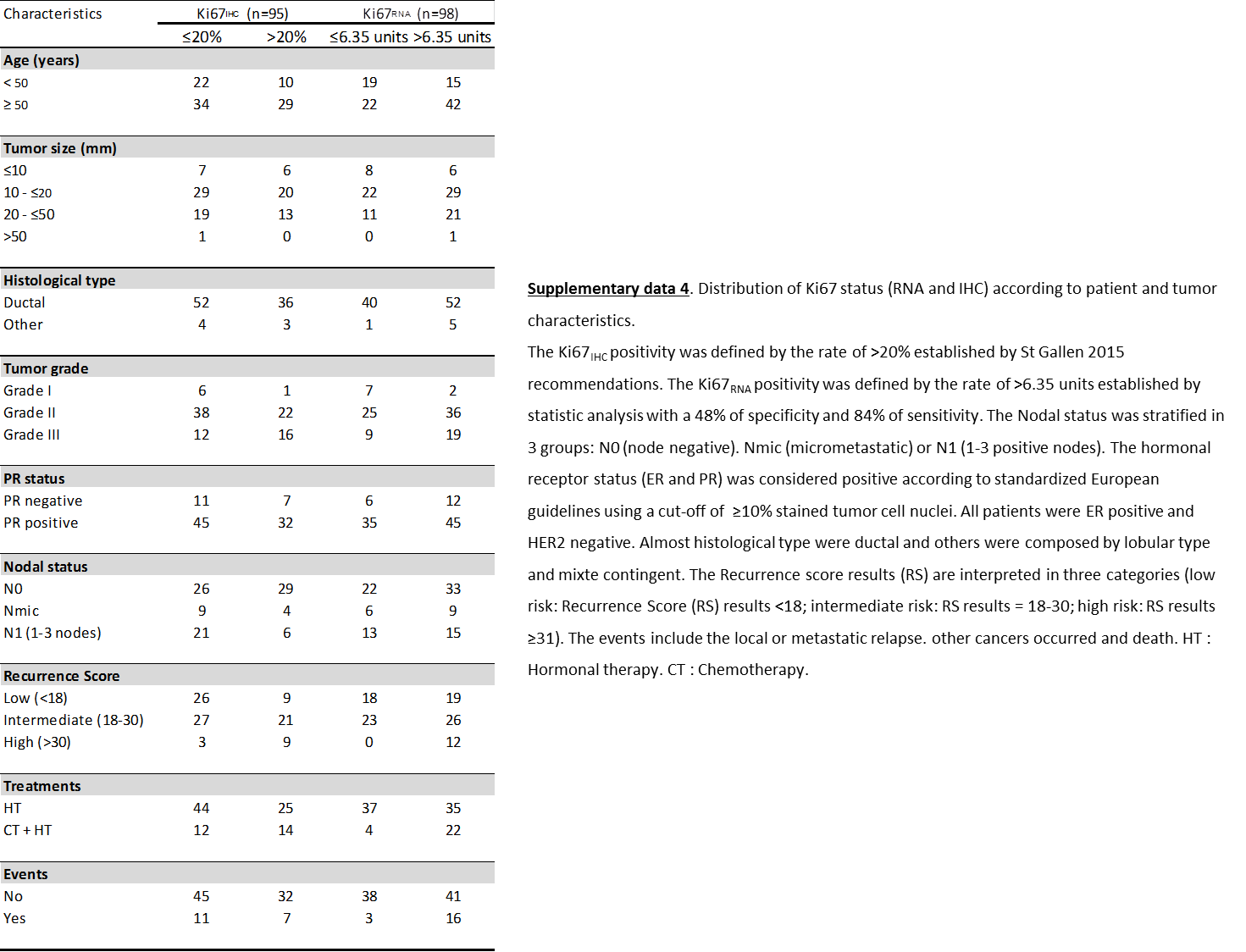

Supplement: Supplementary file 1 — Supplementary Information. [file 41598_2022_7593_MOESM1_ESM.docx]
